# Information Potential Fields Navigation in Wireless *Ad-Hoc* Sensor Networks

**DOI:** 10.3390/s110504794

**Published:** 2011-05-03

**Authors:** Wei Wei, Yong Qi

**Affiliations:** School of Electronic and Information Engineering, Xi’an Jiaotong University, Xi’an 710049, China; E-Mail: qiy@mail.xjtu.edu.cn

**Keywords:** wireless sensor networks, information field, diffusion equation, variation model

## Abstract

As wireless sensor networks (WSNs) are increasingly being deployed in some important applications, it becomes imperative that we consider application requirements in in-network processes. We intend to use a WSN to aid information querying and navigation within a dynamic and real-time environment. We propose a novel method that relies on the heat diffusion equation to finish the navigation process conveniently and easily. From the perspective of theoretical analysis, our proposed work holds the lower constraint condition. We use multiple scales to reach the goal of accurate navigation. We present a multi-scale gradient descent method to satisfy users’ requirements in WSNs. Formula derivations and simulations show that the method is accurately and efficiently able to solve typical sensor network configuration information navigation problems. Simultaneously, the structure of heat diffusion equation allows more flexibility and adaptability in searching algorithm designs.

## 1. Introduction

After the past decade of active research and field trials, WSNs have started penetrating into many areas of science, engineering, and our daily life. They are also envisioned to be an integral part of cyber-physical systems such as those for alternative energy, transportation, and healthcare. In supporting mission-critical, real-time, closed loop sensing and control, the new WSNs represent a significant departure from traditional WSNs which usually focus on open-loop sensing. The stringent application requirements of cyber-physical system (CPS) make it necessary to rethink WSN design.

Earlier applications [1–8] on distributed data collection systems have already identified the merits of cheap networked sensors over traditional centralized sensing systems. Generally speaking, most random individual movement, the simplest of which, the random waypoint mobility model (equivalent to Brownian motion), is adopted to represent pure random movements of the entities of a system [4]. That is why efficient and convenient navigation is important to users. In this paper, we work on the information potential of using a sensor network to aid information querying and navigation through a dynamic and real-time environment. It includes the navigation of packets (responding to client queries from any target node), as well as the navigation of people or automobiles moving in the specified space, such as users with mobile communicating devices seeking to get real-time navigation data with surrounding sensors. The construction and maintenance costs of these information potentials vary according to the high-frequency of queries to the data sources. Most of these gradient-based methods [9–12] utilize the natural gradients of physical phenomena, since the spatial distribution of many physical quantities, such as temperature measurements for heat, follows a natural diffusion law.

The novelty of our method is to establish a practical and convenient information potential field that helps clients to discover the local sub-optimal value points such as free parking spots. Our method can guide the users to reach the local area with higher density of information field instead of one extreme value point. The area of higher density of information field will not attract any conflict because this method navigates the car to the higher density of information area that holds several vacancies for cars. It could avoid lower level competition for parking spots. The role of the heat conduction equation is the time when the resource is updated to meet the physical means in the form of pairs of the information on the degree of each node to be updated. Specially, we imitate an information diffusion process by using a heat equation with specified boundary values. Effectively the information potential sets up a smooth ‘hill area’ (information potential field hill) with several local ‘peaks’; almost all nodes on this area are likely to have several ascending neighbors, and thus greater capacity to reach the different definitions. This trick of smoothing out the discrete hop counts by a heat equation can also be applied in other settings where smooth potential fields of information flow need to be maintained. Finally, we note that others [13] have also used a similar concept motivated by finding the solution to information navigation in sensor networks [8]. In [6,9], the authors use routing based upon an electrostatic potential field and information field; but in those papers the emphasis is on network capacity but not on eliminating competition crises and convenient information discovery, which are the topics explored in this paper.

The rest of the paper is organized as follows. In Section 2, we give the fundamental mathematical formulations of information diffusion and the heat equation. Then, a surface fitting model based on a variation method is introduced and smoothing of a large-scale (global aspect) information field is established. In Section 3, fundamental mathematical formulations of the Laplace equation are described and a continuous small-scale (local aspect) information field is established. In Section 4, several numerical examples are provided. The conclusions are given in the last section.

## 2. Smooth Large Scale Information Potentials

Information resources are changing dynamically from the time the client receives the guidance signal to the time he/she accesses the target node. It is a process where that client accesses the target node after he receives the guidance information. However, in the whole network, information resources are continuously changing dynamically. Therefore, we can make use of a multi-resolution gradient to accomplish this navigation process; that is to say, we can handle different conditions of navigated targets based upon different levels. First, a customer is required to reach the better area with the higher information level, which means to finish the inaccurate navigation or fuzzy guidance. This configuration navigator is a quick and fuzzy process that cannot guarantee the client will reach the specific parking lot, but it can help the customer reach the certain area that holds some extent information field. Then, it could finish the further guidance to get to the assured point. For the fuzzy configuration navigator, we need to build a large scale and smooth information gradient field (global information gradient) based upon the inaccurate gradient descent method.

### 2.1. Information Diffusion and Heat Equation

The initial information gradient field *u*_0_ (*x*, *y*) of the entire network is accurate in every target node. But it cannot reflect the information level of a local area. For the fuzzy configuration navigation, it is necessary for the client to find a field *u*(*x*, *y*) reflecting the local information level. For this local information field, the information is not independent among different nodes but they also affect each other. Consequently, some extent of diffusibility of the information should be considered in the whole network. Every node will expand its own valid information as a source node. The information expanding process is described by a diffusion equation (that is to say, heat flow equation:

(1) $$\frac{\partial u}{\partial t}=c\nabla^{2}\;u$$

**Figure 1.**
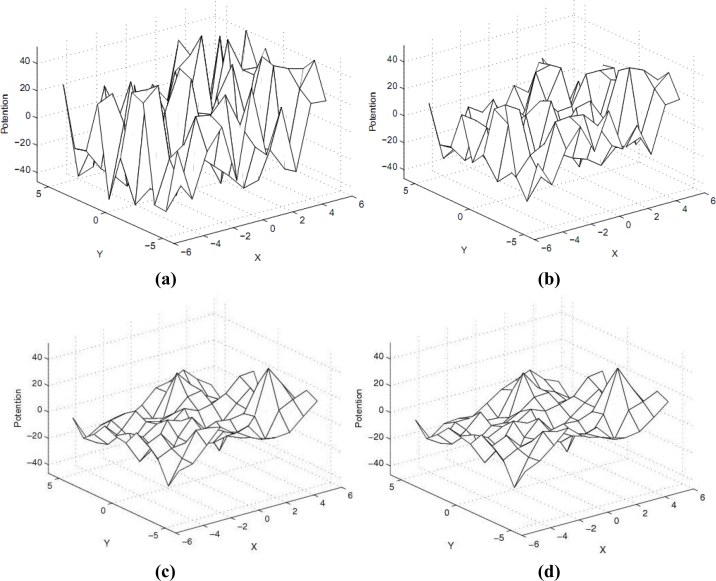
Evolution of information field under the controlling of variable coefficients diffusion equation. **(a)** Initial potential field; **(b)** Potential field at T = 1; **(c)** Potential field at T = 10; **(d)** Final potential field.

In this equation, *c* is Normal number, meaning the proliferation of the speed. As time goes, the original information field gradually becomes a constant field. This result is not useful for resolving the problem. For evolving the information field to a better smooth version preserving the major features, the expanding spreading speed should be controlled and then the following diffusion equation (heat equation) is introduced:

(2) $$\left\{ \begin{array}{c} \frac{\partial u}{\partial t}=F(u)\nabla^{2}u,\\ u(0,\;x,\;y)=u_{0}(x,\;y), \end{array}\right.$$

where u denotes the information potential, *F*_0_ is a positive constant coefficient that represents the information diffusion rate and ∇^2^ denotes the Laplacian operator. As shown in the second term of Equation (2), at time 0, the information potential for any given position (x, y) is a deterministic value. If the information diffusion in a WSN is determined by Equation (1), as time goes by, the information potentials of all positions will finally turn into the same value (See also Figure 1).

### 2.2. Potential Fitting Based on a Variation Method

To get the large-scale smooth information field, the gradient descent method is adopted to navigate the car by the information resource. For the above Section 2.1, it is necessary for us to obtain the more accurate grid and fit the information field layer. Thereby, it is natural to introduce energy functional.

Mesh refinement is performed directly. The four vertexes of each grid hold the specific value, and then it can decide a quadrilateral space. Based on the position inside the defined triangle within the quadrilateral space, the information gradient of the new adding vertex can be calculated based on triangular coordinates.

However, after finishing the refinement, the information gradient surface is still a piecewise triangular plane *u*(*x*, *y*) as before, so that it cannot adopt the gradient method. The balance between accuracy and smoothness defines the following PDE to measure the smoothness of the curved surface and measure the deviation between one surface *v*(*x*, *y*) and the initial surface (the piecewise triangular surface):

(3) $$\begin{array}{l} ɛ(v)=\alpha \int_{\Omega }{\left(v-u\right)}^{2}\;d\Omega +\beta \int_{\Omega }|\nabla v|d\Omega \\ \;\;\;\;\;\;\;\;\;\;\;\;\;\;\;\;\;\;\;\;\;\;\;\;+\gamma \int_{\Omega }{\left|\nabla v\right|}^{2}\;d\Omega \end{array}$$

In the above functional, *α* and *β* are normal parameters. To get an optimal surface v^*^ (*x*, *y*) (this surface is smooth enough and the deviation between it and *u*(*x*, *y*) is small enough), the following optimal problem can be built up.

It is needed to search v^*^ (*x*, *y*) that satisfy $ɛ(v^{*})=\text{min}\{ɛ(v)|v\in H_{0}^{1}(\Omega )\}$ where $H_{0}^{1}(\Omega )$ the Soblev space of is Ω. Consequently, we compute the Euler-Lagrange equation of energy functional (3), and then we introduce the additional part $\frac{\partial u}{\partial t}$ so that the corresponding evolution equation is obtained as follows:

(4) $$\left\{ \begin{array}{c} \frac{\partial u}{\partial t}=-2\alpha (v-u)+\beta \text{div}(\frac{\nabla v}{\left|\nabla v\right|})+2\gamma \nabla^{2}v,\\ v(0,\;x,\;y)=u(x,\;y), \end{array}\right.$$

**Figure 2.**
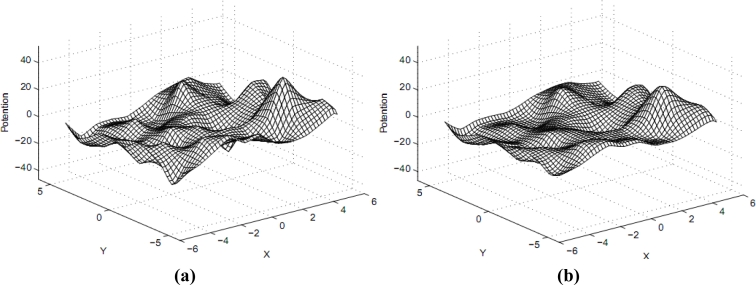
Evolution of potential field under the controlling of variable coefficients diffusion equation. **(a)** Potential field at T = 0.1; **(b)** Final potential field.

Set *α*= 1, *β* = 1, *γ* = 5, *τ*= 0.01 and set the relative error limit as 0.01%. The central-difference scheme is used in computation and a smooth large-scale potential field (see also Figure 2) can be obtained after 478 iterations. After finishing construction of the large-scale smooth information potential field *v*(*x*, *y*), we can utilize the gradient descent method to navigate clients.

## 3. Small Scale Information Potentials Based on LAPLACE Equation

By the fuzzy configuration navigation information of the large-scale information field, the client reached the higher information field level, and then, the customer needs to be navigated accurately within the small-scale (local) information field. Finally, the client could reach a certain node and finish the demand. To finish the final configuration navigation object, smooth information is built up and it is accurate on each local node. The result of Laplace equation boundary value problem is helpful for this goal:

(5) $$\left\{ \begin{array}{cc} \nabla w^{2}=0, & (x,\;y)\in D,\\ w(0,\;x,\;y)=v(x,\;y), & (x,\;y)\in \partial D, \end{array}\right.$$

**Figure 3.**
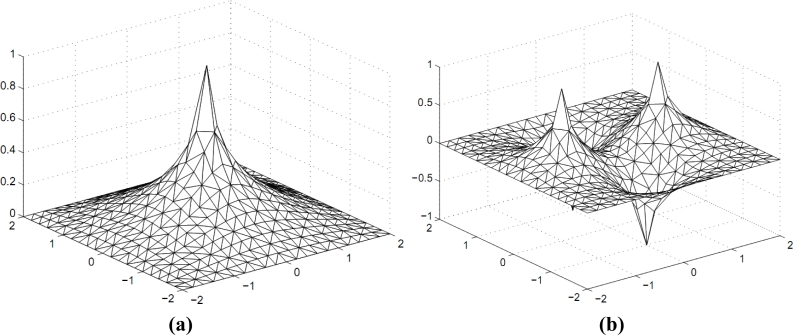
Results of Laplace problems in several non-convex fields.

It is acknowledged that the value of any internal point can be determined uniquely if the values of the information field function on the boundaries of the non-convex area are given. In addition, based on the maximum principle, certain extreme points must lay on the boundary. We are enlightened by the previous work of Gao (see [13–15]; they provide a method that set up a smooth information field based on the limited boundary conditions). Therefore, a small-scale method is established. Figure 3 shows several Laplace problem examples on the non-convex field.

Consequently, we can establish a similar Laplace problem in the current node where the local area lies so that we get the small-scale information potential field *w*(*x*, *y*)(in the local node, the value is accurate but not smooth) that is almost smooth everywhere. After computation, then the current node will be updated as the local maximum point of the small-scale potential field *w*(*x*, *y*). This node must be one of the nodes of the original discrete information potential field *u*_0_ (*x*, *y*) that satisfy the requirement of the information level.

## 4. Multi-Scale Gradient Descent Method

In this section, by integrating the above mentioned, the process of the multi-scale gradient descent method will be illustrated as follows:

1. According to the current user’s requirement, the information boundary of the local area of the target node can be set.
2. Based on the current sensor networks, in terms of the Section 2, the large scale information potential field can be obtained based on the heat equation. Simultaneously, the certain target with the satisfied information level can be reached via the gradient descent method.
3. From the current user’s node, in terms of the Section 3, the local small scale information potential field can be obtained, thereby; we can reach the node that makes the client satisfactorily depend on the gradient descent method.

**Figure 4.**
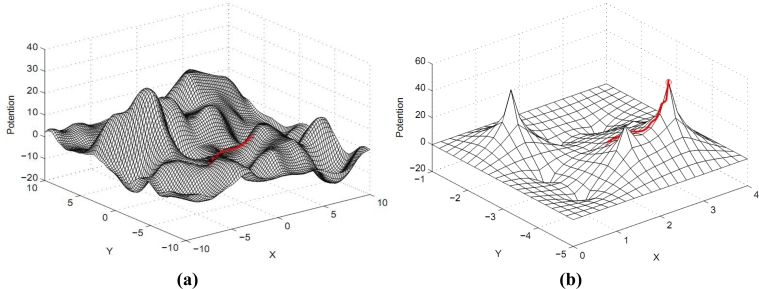
Multi-scale gradient descent process of single user. **(a)** Large scale gradient descent; **(b)** Small scale gradient descent.

The following two examples are given to verify the validity of this method:

Example 1. Single customer requirement. We randomly generate a user node in the network, and start navigation to lead users to reach the destination node which satisfies the information level boundary. The multi-scale gradient descent process of shown in Figure 4. Through both theoretical and simulation analysis, we find that multi-scale gradient descent method can efficiently decrease the level of the competition as several clients intend to move forward to one spot, etc. From the mathematical theoretical perspective, the original work [13] not only requires the second derivative must exist but also needs to satisfy the Laplace equation. Our method only requires constructing the potential field to satisfy the first-order continuous differentiable as shown in Equation (4). The constraint condition of constructing the potential field is obviously decreased. It is very important when defining the structure of the internal relationship between clients and information nodes. In Figure 7, we can clearly verify that the users usually move forward the lower information level area based on the previous method. Meanwhile, it will cause the fierce competition collisions since the idle spots are less numerous than the number of users. Our means can eliminate competition as far as possible and finish the navigation conveniently. The greatest advantage of our work is that it can coordinate the demand relationship of clients and decrease the total collisions.

**Figure 5.**
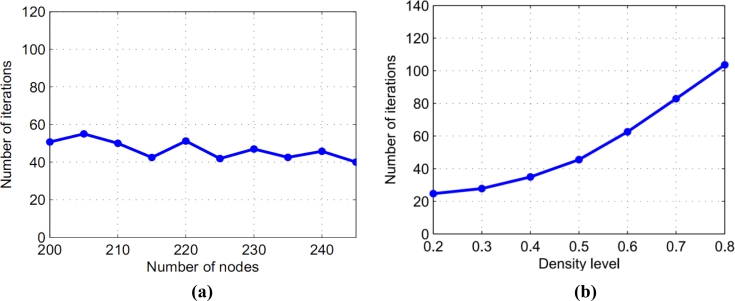
Relation about density and the numbers of iterations, nodes. **(a)** At the density 0.5; **(b)** Different densities.

Example 2. Several users’ requirement. Some clients are randomly generated in the network and competition is considered. The large scale gradients descent process and the final results of navigation are shown in Figure 5.

## 5. Discussions

We estimate information potential fields by simulation in the following aspects: the construction and maintenance costs of the information potential field, robustness to network, the balance of query qualities versus gradient precision cost, as well as the applications of the potential fields in Section 3.

### 5.1. Simulation Setup

We simulate wireless transmission using ordinary mode in the same radio models of TOSSIM. In the ordinary mode, all nodes within the transmission range can communicate fluently under ideal conditions. We feed our node locations in the TOSSIM radio model and obtain connectivity and link quality for each pair of nodes. At any time slot we can set a percentage of randomly selected links to be not available, throughout all the experiments. The maintenance of the information field is on-demand. We establish the gradient on a neighbor discovery protocol. Gradient maintenance and routing are in the networking layer, and can be integrated with existing protocols that maintain a neighbor list for each node [15].

### 5.2. Robustness to Potential Construction

Suppose we have a perturbed grid network of size 20 × 20 nodes and consider establishing smooth potential fields with different conditions. First, we generate some random nets which have different topologies and different nodes numbers but have the same density. The numbers of nodes are between 200 and 245. The numbers of iterations for different nets are shown in Figure 5(a). Next, we generate random nets which have different densities. The numbers of iterations for different nets are shown in Figure 5(b). The result of the simulation shows that our method is robust to establish smooth potential fields for sensor networks.

### 5.3. Avoidance of Competition

**Figure 6.**
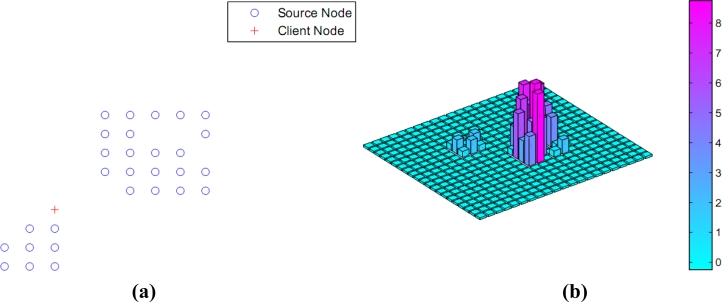
Topology and histogram. **(a)** Nodes topology; **(b)** Information histogram.

What’s more, our method can coordinate the requirements of different users and the competition collisions can be reduced or even eliminated. As shown in Figure 6, one user flagged by a red ‘+’ was at (−2,–2), there are two reasonable directions (the lower left and the upper right directions) to reach the destination that satisfied the user’s requirement.

**Figure 7.**
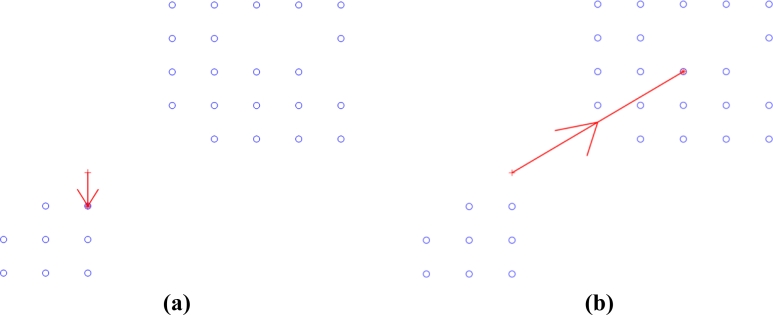
Navigation results of different methods. **(a)** Result of previous method; **(b)** Our result.

If there is no competition, the lower left moving direction has a closer distance to the users so that it has smaller cost, and *vice versa*. However, in a real scenario, when competition happens, users need to start navigation again and move forward the upper right direction, so that causes the bigger cost. We can find the moving possibility is closely affected by the level of competition and the quantity of information. Generally speaking, moving direction is inversely proportional to the information quantity and proportional to the fierce level of competition. The previous work [13] only leads to the result of Figure 7(a) and cannot avoid the competition collisions. The display of Figure 7(b) is to further supplement and highlight that of the Figure 7(a).

**Figure 8.**
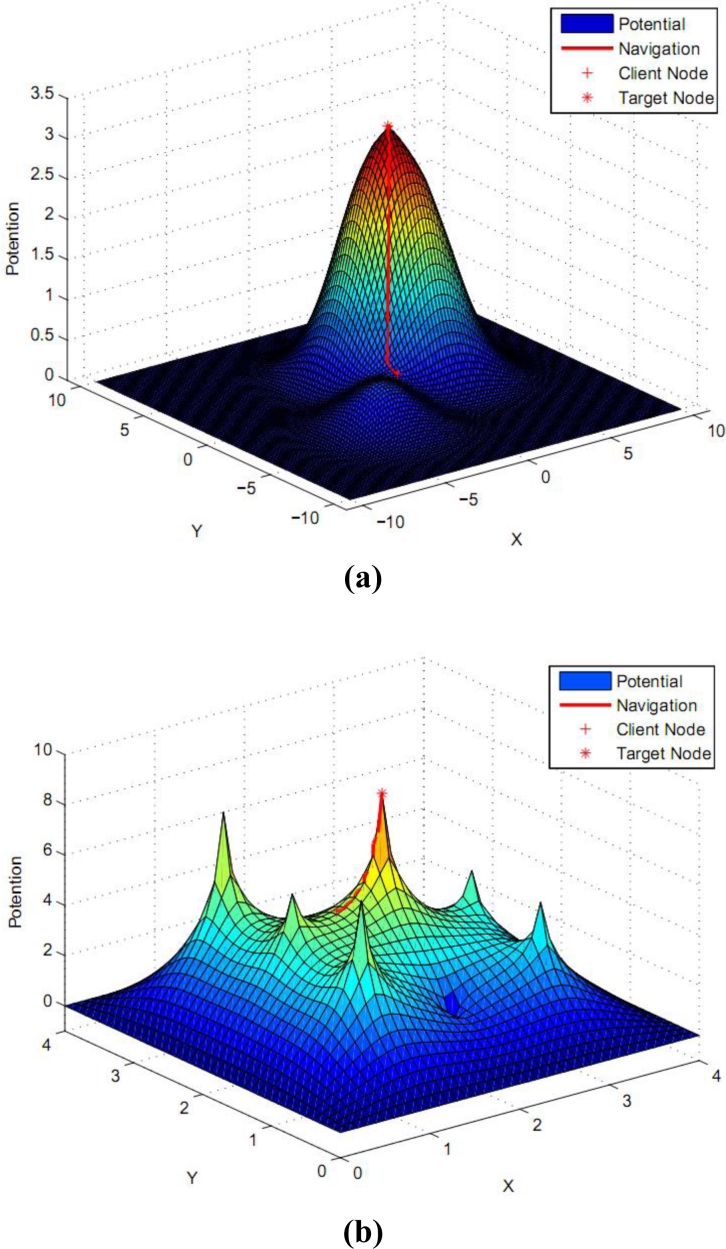
Multi-scale gradient descent of one user. **(a)** Guided to a region with high level; **(b)** Guided to a certain node with satisfied information.

Since Figure 7(a) is a plane one that features unclear demonstration, Figure 7(b) displays the navigation results of Figure 7(a) in the right picture in a information potential field with a three-dimensional figure. Red color indicates an area of high potential information, which shows that our navigation system will guide the customers to the upper right place after they launch a query. In the Figure 7(b), the blue area indicates a lower amount of information; therefore the altitude is lower correspondingly, and *vice versa*. The main reason is that the earlier method independently thinks over the information of different directions that has closer connections and distinctions. However, our work more carefully considers the above intrinsic connection and differences so that we can obtain the better result shown in Figure 7(b). Thereby, our work can efficiently avoid the competition and reduce the cost. The navigation result of multi-scale gradient descent process is seen also in Figure 8. Figure 8 generally shows that after users send out a query; our approach will lead them to a higher information potential area.

As Figure 8(a) shows, the higher information field region users arrive at looks like a single point. (in accordance with the old way, it is an accurate point.) But according to our approach, this so-called single point can be enlarged and found to be an area which holds lots of information points with large amount of higher level information. It also means this region contains amounts of higher information spaces for users’ selection.

### 5.4. Navigation Example

**Figure 9.**
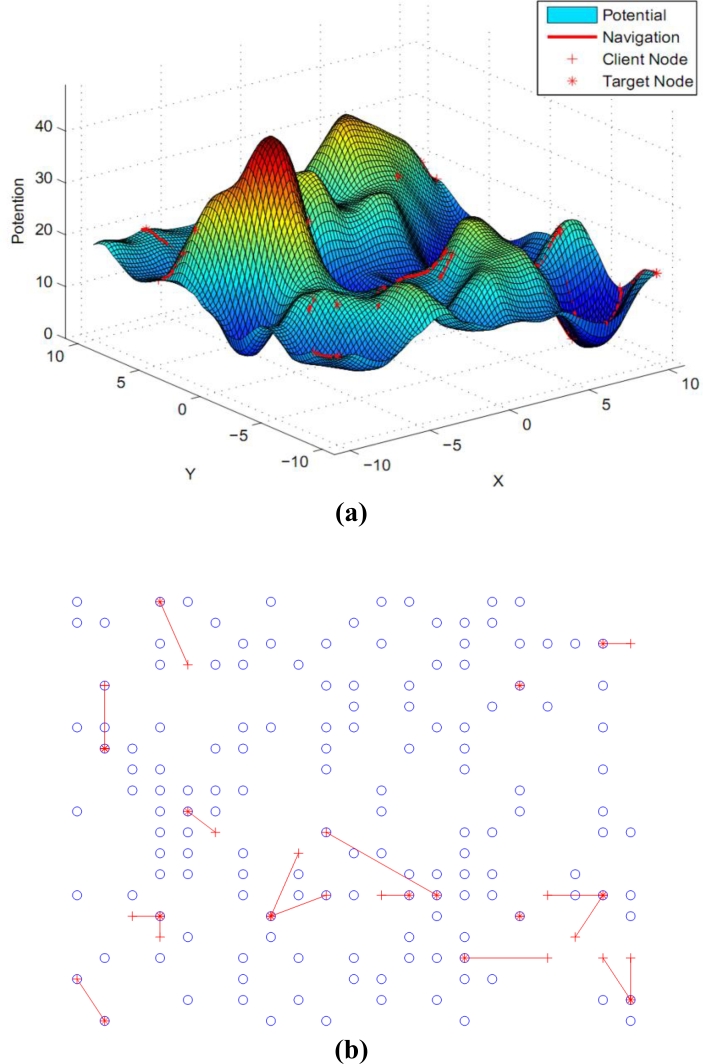
Navigation results of several users. **(a)** Guided to the regions; **(b)** Topology of the navigation.

Several users’ requirement. Twenty users are randomly generated in the network and competition is considered. The large scale gradient descent process and the final results of navigation are shown in Figure 9 and Table 1. The results show that all the users finally arrive at a target node which has high information level. This means that the adverse effects of competition have been reduced to a very low level.

## 6. Conclusions

In this paper, we have proposed a brand new heat diffusion equation to finish the navigation process conveniently and easily. Partitioned scales are used to reach the goal of the accurate navigation. Some theoretical tools such as heat diffusion equation, PDE variational method, and gradient descent methods are adopted in our method. Two smooth potential fields of sensor network are helpful to satisfy the customers’ requirement. Multi-scale gradient descent methods, examples and solid mathematical principles show that the method is accurate and efficient, able to solve typical sensor network configuration information navigation problems. The nonlinear PDE structure allows more flexibility and adaptability in searching algorithm designs.

Compared with the former works depending on the discrete information field, our method ensures a local information field large enough to include appropriate multiple targets and the competition conflicts can be resolved simultaneously. The information level of each node can be updated with a satisfactory physical methodology when resources are dynamically changing. By developing an algebraic structure of heat diffusion equation, we can combine different potentials to enable far greater path diversity and thus provide better performance than it is possible with only one-fold discrete field guidance. The simulation results show that although with much relaxed assumptions, our approach achieves comparable performance with significantly reduced competition collisions. We will further explore this direction in the future.

## Figures and Tables

**Table 1.** Navigation results.

**Current level**	**Current node**	**Target node**	**Target level**
−1.5261	(−1, −1)	(−4, 3)	54.4739
76.4739	(−6, −9)	(−6, −9)	76.4739
−17.5261	(−7, −6)	(10, −7)	61.4739
−17.5261	(−7, 9)	(−9, 10)	46.4739
−17.5261	(−6, −7)	(−5, −7)	82.4739
−17.5261	(−4, 7)	(−4, 9)	70.4739
−17.5261	(−4, 1)	(−4, 2)	33.4739
−17.5261	(−8, 10)	(−8, 9)	80.4739
70.4739	(−3, −9)	(−3, −9)	70.4739
35.4739	(−4, −1)	(−4, −1)	35.4739
−17.5261	(−5, −8)	(−5, −7)	82.4739
−17.5261	(−7, 10)	(−9, 10)	46.4739
49.4739	(−8, −10)	(−8, −10)	49.4739
73.4739	(−5, 6)	(−5, 6)	73.4739
−17.5261	(−7, 7)	(−6, 4)	37.4739
−17.5261	(−6, 8)	(−4, 9)	70.4739
76.4739	(−6, −9)	(−6, −9)	76.4739
−17.5261	(−2, −2)	(−5, −3)	56.4739
−17.5261	(−1, −5)	(−0, −6)	78.4739
58.4739	(−6, 6)	(−6, 6)	58.4739
